# Endocrine immune-related adverse events in patients with metastatic renal and urothelial cancer treated with immune checkpoint-inhibitors

**DOI:** 10.1007/s11255-023-03635-9

**Published:** 2023-06-05

**Authors:** Immanuel Augustin Oppolzer, Josef Riester, Roland Büttner, Maximilian Burger, Marco Julius Schnabel

**Affiliations:** 1 Department of Urology, University of Regensburg, Caritas St. Josef Medical Center, Regensburg, Germany; 2 Department of Internal Medicine Caritas St. Josef Medical Center, Regensburg, Germany

**Keywords:** Kidney Cancer, Bladder Cancer, immune-related adverse events, Hypophysitis, Checkpoint inhibition, PD-1, PD-L1, CTLA-4

## Abstract

**Purpose:**

To evaluate the incidence, diagnosis and treatment of immune-related adverse events (e-irAE) of checkpoint inhibition (ICI) in metastatic urothelial carcinoma (mUC) and metastatic renal cell carcinoma (mRCC).

**Methods:**

A retrospective, single-center study was conducted to identify a cohort that received ICI for mUC or mRCC. e-irAE were classified according to the CTCAE V.5.0. Patients received ICI for mUC or mCC between 01/2017 and 03/2021. A retrospective chart review was performed. T-Test, the chi-squared test, and Fisher's exact test were performed.

**Results:**

102 Patients received ICI [mUC: 40 (39%), mRCC: 62 (61%)]. 64 (63%) received an ICI monotherapy, 27 (27%) a dual ICI therapy, 11 (11%) a combination with VEGFi. e-irAE occurred in 19 (19%) patients [grade 1–2: 17 (84%), grade 3: 3 (16%)]. The median time until e-irAE was 42 days (range 11–211 days). 14 Patients developed thyroidism (14%), 4 (4%) a hypophysitis, 1 (1%) an adrenal insufficiency (AI). 7 patients (7%) had to discontinue ICI therapy [hypophysitis (100%), AI (100%), thyroidism (14%)]. 6 (86%) received cortisone. After a median range of 34 days 5 patients (71%) restarted ICI therapy. All patients (n = 4) with hypophysitis continued ICI [4 (100%) prednisone, 3 (75%) levothyroxine]. 11 (79%) presented with hyperthyroidism. 4 (37%) needed therapy (1 (7%) prednisone, 3 (21%) thiamazole, 2 (14%) beta blocker). The 9 (64%) patients with hypothyroidism received levothyroxine. Hypophysitis appears only on dual ICI (CTLA-4/PD-1) inhibition (p 0.007).

**Conclusion:**

This study shows the importance of adequate diagnosis and therapy of e-irAEs.

## Introduction

The treatment of uro-oncological cancer with immune checkpoint inhibitors (ICI) has been established for urothelial carcinoma and renal cell carcinoma since 2016 and has led to a significant improvement in the prognosis [1, 2]. The most frequently used agents are directed against “Programmed cell death protein 1” (PD-1), “Programmed cell death 1 ligand 1” (PD-L1) and “Cytotoxic T-lymphocyte antigen 4” (CTLA-4) [3, 4]. The toxicity profile of ICI is favorable compared to standard of care, yet immune-related adverse events (irAE) could affect any organ and might require rapid diagnosis and intense clinical management [5]. The probability of any grade irAE is approximately 70% with ICI monotherapy; a dual ICI regime increases the risk, especially of high grade irAE [6]. The most affected organs are the gastrointestinal tract, endocrine glands, skin, and liver [7]. Generally, CTLA-4 inhibitors appear to cause more serious side effects [8]. Usually, the irAE occur during the first months on treatment, but individual cases with late-onset irAE were reported even after ICI cessation [9]. The endocrine irAE include inflammation of the thyroid, endocrine pancreas, adrenal and pituitary glands [10]. Because hyperthyroidism often evolves into hypothyroidism, changes in the function of the thyroid gland are usually summarized as thyroidism [10]. Thyroid dysfunction occurs more frequently during therapy with PD-1- and PD-L1-inhibitors. Hypophysitis is more likely to occur during therapy with CTLA-4-inhibtiors [11]. Insulin-dependent type 1 diabetes mellitus and primary adrenal insufficiency (AI) are rare side effects [7, 11]. This study examines the endocrine immune-related adverse events (irAEs) associated with ICI treatment in patients with metastatic urological cancer. The study is based on a cohort of patients with metastatic urothelial or renal cell carcinoma who were diagnosed and treated in a large uro-oncological centers of Germany.

## Methods

This single center study encompasses 107 patients who received ICI for metastatic urothelial or renal cell carcinoma between 01/2017 and 03/2021. A retrospective chart review of patients who received immunotherapy using CTLA-4 (Ipilimumab) and/or PD-1 (Nivolumab, Pembrolizumab) or PD-L1 (Atezolizumab, Avelumab) antibodies in the uro-oncological department at the University of Regensburg was performed. Routine clinical exams included a questionnaire-based screening for typical adverse event related symptoms. Routine lab parameters screened for endocrine dysfunction were electrolytes (Na, K, Ca), thyroid-stimulating hormone (TSH), free thyroxine (fT3, fT4), glucose levels, and morning serum cortisol (8 a.m.).

Patients with suspicious routine lab work underwent an endocrine irAE workup with [10, 12]:

- TPO (thyroid peroxidase) antibodies if TSH is high
- TRA (thyrotropin receptor antibodies) antibodies if TSH is low
- Luteinizing hormone (LH), follicle-stimulating hormone (FSH), testosterone, estrogen in premenopausal women, adrenocorticotropic hormone (ACTH) if hypophysitis or adrenalitis is suspected.
  o MRI of the brain if symptoms are present
  p Short-Synacthen-Test (SST)
  q Adrenalitis: plasma aldosterone and renin levels.
- HbA1c for elevated glucose
  o serum/urine ketones and venous blood gas
  p C-peptide and anti-islet cell antibodies

Toxicities were assessed using the “Common Terminology Criteria for Adverse Events” (CTCAE V.5.0.). Newly diagnosed hyper- or hypothyroidism, hypophysitis, type-1 diabetes mellitus, or AI were rated as endocrine, treatment-related irAE. In the case of hypophysitis, the dysfunction of the thyroid gland was not listed as a separate irAE.

### Statistical analysis

SPSS 28.0 (SPSS Inc., Chicago, IL) was used T-test, chi-squared test and fisher’s exact test.

### Ethical and legal aspects

This study was carried out in accordance with The Code of Ethics of the World Medical Association (Declaration of Helsinki). All members of the research team committed themselves to the confidentiality of the information provided as well as to data protection and are subject to medical confidentiality.

## Results

### Patient characteristics

As shown in Table 1, a total of 107 patients received ICIs between January 2017 and March 2021. 5 patients were excluded because of missing follow-up data. 40 (39%) patients had a mUC, 62 (61%) had a mRCC. The cohort consisted of 30 (29%) women and 72 (71%) man. The median age was 66.4 years (range 40–90). 64 (63%) patients received an ICI monotherapy, 27 (27%) a dual ICI therapy, and 11 (11%) a combination with the VEGFR-inhibitor Axitinib. The majority (88%) received a PD-1 based therapy, whereas 14% were treated with a PD-L1 based regimen. 55 (54%) received ICI-therapy as a first line treatment. 47 (46%) had one or more treatment lines in advance.

**Table 1:** Patient’s characteristics

Patient’s characteristics (N = 102)	Patient, n (%)
	Total (N = 102)
Age at start	
Median (min, max)	66.8 (40–90)
Sex	
Male	72 (70.6)
Female	30 (29.4)
Line of ICI	
First	55 (53.9)
≥ Second	47 (46.1)
Type of ICI	
PD-1 inhibitors	88 (86.3)
Nivolumab	53 (51.9)
Pembrolizumab	35 (34.2)
PD-L1 inhibitors	14 (13.7)
Atezolizumab	13 (12.7)
Avelumab	1 (1.0)
ICI type	
ICI monotherapy	64 (62.7)
ICI + CTLA-4 (ipilimumab)	27 (26.5)
ICI + VEGFi (axitinib)	11 (10.8)

*ICI* immune checkpoint inhibitors

### Characteristic and incidence of immune-related endocrine adverse events

As shown in Table 2, any grade endocrine immune-related adverse events (irAE) occurred in 19 (19%) patients, all of them developed endocrine irAEs while on treatment. IrAE were mostly grades 1–2 (84%), grade 3 occurred in 16%, and no one developed grade 4–5 irAE. The most frequent irAE were thyroidism (n = 14, 73%) and hypophysitis (n = 4, 21%), AI occurred in 1 case (5%). No patient developed a type-I diabetes mellitus. The median time until the experience of an endocrine irAE was 42 days (range 11 – 211 days). All endocrine irAE occurred within the first 12 months, 7 (37%) during the first month on therapy, 10 (53%) within the first 3 months, 2 (11%) within 12 months and none thereafter, respectively. Patients diagnosed with hypophysitis (n = 4) presented with decreased serum sodium in three of four (75%), increased serum potassium in two (50%), decreased TSH in three (75%), increased TSH in one (25%), decreased fT4 in two patients (50%) and reduced cortisol and ACTH in all patients, respectively. A brain MRI was performed in one of four patients (25%). No pathologies were seen. The clinical symptoms were non-specific and included fatigue, dizziness, and headache. No patient reported impaired vision. All nine patients with hyperthyroidism presented with changes in TSH and fT3 or fT4. Four (44%) of them were symptomatic (restlessness, sweating, tachycardia, insomnia) and required intensified therapy with thiamazole, β-blockers and one with corticosteroids. The patient with AI complained of fatigue (CTC II). Routine lab work revealed decreased serum sodium with slightly increased serum potassium and a low glucose level. The diagnosis was confirmed by low cortisol level with slightly elevated ACTH and by SST.

**Table 2:** Endocrine adverse events

Endocrine adverse event	All grade, n (%)	CTC grade 1–2	CTC grade 3–4
	Total (N = 102)	n (%)	n (%)
All	19 (18)	16 (84)	3 (16)
Thyroidism	14 (13.6)	14 (100)	
Hypophysitis	4 (3.8)	2 (50)	2 (50)
Adrenal insufficiency	1 (0.97)		1 (100)

*CTC* common terminology criteria

### Endocrine irAEs: treatment, ICI interruption and ICI restart

7 of 19 patients (37%) with endocrine irAE had to interrupt ICI therapy, including all patients who developed hypophysitis or AI but only two of 14 with thyroidism. Six of these seven patients (86%) with ICI interruption received cortisone-based therapy. After a median range of 34 days five patients (71%) restarted the ICI therapy. One (14%) died due to unrelated comorbidity. One (14%) experienced a complete response of the metastasis and underwent resection of the remaining primary tumor. Afterwards the patient switched to a surveillance protocol without any systemic treatment, 11-month-follow-up is unremarkable. All patients (n = 4, 21%) with hypophysitis were restarted on ICI with ongoing substitution of 5 mg prednisolone, and three (75%) receive levothyroxine additionally. 11 (78%) of 14 patients with thyroidism initially presented with hyperthyroidism. Four (37%) of them received symptomatic treatment, one (7%) with prednisolone, three (21%) with thiamazole and two (14%) with a beta blocker additionally. Two (14%) had no symptoms and did not receive specific therapy. So far, four (28%) of the patients with hyperthyroidism didn’t develop hypothyroidism. All ten (71%) patients with hypothyroidism (3 primary, 7 secondary) received L-thyroxin substitution.

The only patient who experienced adrenalitis and secondary adrenal insufficiency is on substitution therapy with 5 mg/d prednisolone. An additive substitution with 0.1 mg/d fludrocortisone was stopped on further course.

### Risk factors for developing an immune-related endocrine adverse event

As shown in Table 3 the occurrence of endocrinopathy revealed a significant impact of first-line therapy (p = 0.013) and the combination of PD-1 with CTLA-4 therapy vs. monotherapy (p = 0.007). Endocrinopathies occurred in patients receiving PD-1-inhibitors (19/88 patients), but not PD-L1-inhibitors (0 /14 patients). No statistical significance was seen for the individual side effects (hypophysitis, thyroiditis, adrenalitis) related to PD-1 vs. PD-L1. Neither sex nor age had a statistically significant influence on the occurrence of endocrinopathy. Hypophysitis only occurred with CTLA-4 inhibitor-based therapy, but not in CPI therapies without (p = 0.007). There was no significant difference in the occurrence of thyroidism related to gender or the therapy modality used, even if combination therapy (PD-1 +CTLA-4 or VEGFi) generally seems to be associated with a higher risk of occurrence (data not shown).

**Table 3:** Risk Factor for irAE

Risk factor	Endocrinopathy	No endocrinopathy	p value
	n (%)	n (%)	
Entity			
Urothelial carcinoma	5 (9.4)	48 (90.6)	0.202^+^
Renal cell carcinoma	14 (38.9)	22 (61.1)	
Lines of therapy			
First line	15 (27.3)	40 (72.7)	0.013^#^
Second or more line	4 (8.5)	43 (91.5)	
Age [mean]	66y (n = 19)	66y (n = 83)	0.974*
Gender	Female: 8 (26.7)	Female: 22 (73.3)	0.178^+^
	Male: 11 (15.3)	Male: 61 (84.7)	
Therapy type			
PD-1	19 (21.5)	69 (78.5)	0.328^#^
PD-L-1	0 (0.0)	14 (100)	
Therapy type			
PD-1 or PD-L-1 (mono)	7 (10.9)	57 (89.1)	0.014^+^
PD-1 + CTLA4	10 (37.0)	17 (63.0)	
PD-(L)-1 + VEGFRi	2 (18.2)	9 (81.8)	
Therapy type			
PD-1 or PD-L1 (mono)	7 (10.9)	57 (89.1)	0.612^#^
PD-(L)-1 + VEGFRi	2 (18.2)	9 (81.8)	
Therapy type			
PD-1 or PD-L1 (mono)	7 (10.9)	57(89.1)	0.007^#^
PD-1 + CTLA4	10 (37.0)	17 (63.0)	
Therapy type			
PD-(L)-1 + VEGFRi	2 (18.2)	9 (81.8)	0.444^#^
PD-1 + CTLA4	10 (37.0)	17 (63.0)	

Prevalence of endocrinopathy is independent of age and gender but affected by the therapy line and type of ICI-therapy

*T test

^#^Fishers exact test

^+^Chi-square test

## Discussion

Since the first game-changing trial of ICI-therapy in urothelial cancer [1], ICI-therapy has evolved into an essential part of the systematic therapy of urothelial and renal cell carcinomas [13]. Highest attention and a standardized irAE screening are required to diagnose ICI-induced endocrinopathy, as the presentation is often non-specific, like fatigue, nausea, headache, or weakness. Therefore, diagnosis of irAE endocrinopathies might be delayed or treatment insufficient, which can result in serious health conditions [14]. Higher rates of endocrine side effects under combination therapy than in monotherapy, especially immune checkpoint combination therapy, are known in the literature [6, 15, 16]. Our cohort reflects this higher incidence with 11 (+CTLA-4) vs. 3 (+VEGFR) vs. 5 (mono) in PD1 + CTLA-4, PD-(L)1+VEGFR and PD-(L)1, respectively. Not just the occurrence, but also the AE grade is known to be higher in CTLA-4-based therapies than in PD-1/ PD-L1 based therapy [15]. As well, this is supported by our cohort with all grad 3/4 side effects in patients with CTLA-4 based therapy. The need for lifelong hormone replacement is another peculiarity of endocrine irAEs [5], which was necessary in 73% of our patients.

For a good outcome, timely diagnosis and initiation of therapy are essential. There are different recommendations for structured monitoring of patients receiving immunotherapy to detect autoimmune-mediated side effects in a timely manner. The routine monitoring protocol of the NCCN Guidelines for Management of Immunotherapy-Related Toxicities [10] recommends a baseline assessment at each visit to evaluate any abnormal findings in physical examination, patient history, endocrinopathy, infectious disease, neurological examination, and bowel habits. CT imaging and brain MRI should be performed periodically if indicated. Laboratory analysis of TSH and free thyroxine is recommended every 4–6 weeks during immunotherapy for thyroid irAEs, with total T3 and TPO antibodies tested if TSH is high and TRAbs if TSH is low. For adrenal/pituitary irAEs, serum cortisol, TSH, and free T4 should be tested every 2–3 weeks during immunotherapy, then followed up every 6–12 weeks. Comprehensive metabolic panels should be conducted every 2–3 weeks during immunotherapy to monitor for diabetes mellitus, with HbA1c tested for elevated glucose levels. Stelmachowska-Banaś et al. [20] propose a similar routine monitoring protocol with clinical examination and laboratory tests, which include sodium, potassium, calcium, TSH, fT4, and serum cortisol at 8 am before initial administration, then before every drug infusion for 6 months, then every 2–3 months for the next 6 months and thereafter every 6 months. This protocol differs from the NCCN Guidelines for Management of Immunotherapy-Related Toxicities in terms of intervals for laboratory tests during treatment become larger in this protocol, whereas in the NCCN guidelines, the time intervals for laboratory analyses remain the same throughout the entire treatment. A German study group [12] focuses on irAEs and proposed an evaluation of clinical history, blood pressure, fluid intake, glucose, sodium, potassium, creatinine, cortisol, and ACTH as well, if indicated, testosterone for male, estradiol for female patients and FSH and LH before initial administration and thereafter before every drug infusion, but at least every 4 weeks. In premenopausal women the sex hormones do not need to be measured. The analysis of the sex hormones testosterone and estradiol as baseline analysis before initial administration without suspicion of AI or hypophysitis is unique in this recommendation and it is not recommended to repeat them if there are no abnormalities. The NCCN recommendation suggests the most frequent laboratory analysis. Our routine monitoring is closest to the one from the German study group of Mai et al. [12], with routine clinical exams for typical irAEs and routine lab parameters screened for endocrine dysfunction (Na, K, Ca), TSH, fT3, fT4, glucose levels, and morning serum cortisol at 8 a.m. before initial administration and before every subsequent administration. For the first 3 months of immunotherapy we perform an intensified lab examination at least every 4 weeks, even if the therapy intervals are longer.

The subsequent sections aim to evaluate specific irAEs and provide recommendations for their corresponding diagnosis and treatments.

### Hypophysitis

On average, the side effects occurred about 2.5 months after the start of therapy. This is in line with the literature (1.75–3 months) [11]. The prevalence of combined CTLA-4 + PD-1 therapy was higher (14%) in our study than previously reported (8.5–9%) [17]. We were able to confirm that hypophysitis is more likely to be associated with CTLA-4-inihibitor therapy, which is consistent with the literature [6, 15]. Solinas et al. were able to show that the occurrence of hypophysitis depends on the dose of Ipilimumab [18]. A possible explanation is the physiological CTLA-4 expression in normal pituitary tissue, which might support the binding of CTLA-4 antibodies and result in hypophysitis [19]. The increased incidence of hypophysitis described among men was not seen in our analysis [19]. However, this might be due to the small sample size of our trial. Given the non-specific clinical symptoms (weakness, nausea, appetite-loss, cold-intolerance) laboratory tests are important. These should include fasting glucose, electrolytes, TSH, fT4, and early morning cortisol levels. Patients with low cortisol levels (≤ 18 μg/dL) should be assessed for plasma ACTH. Additionally, testosterone, LH, and FSH levels should be assessed in males, FSH levels in postmenopausal women, and estradiol, LH, and FSH levels in premenopausal women with irregular menstruation [12, 20].

Although the MRI often shows a mild to moderate swelling of the hypophysitis in the context of a CTLA-4 induced hypophysitis, the MRI is more valuable in the exclusion of a differential diagnosis (metastasis, primary hypophysitis). Clinical management should focus on symptoms [9, 16].

While standard treatment of irAE is based on immunosuppression with glucocorticoids, the ideal dosing is still under debate [6]. An initial high-dose therapy is favored by NCCN-Guidelines. Faje and colleagues showed a survival advantage and a longer time until therapy failure in a large retrospective study for lower glucocorticoid doses (< 7.5 mg prednisolone equivalent/ d) [21]. Stelmachowska-Banaś and colleagues recommend high-dose glucocorticoids only in selected patients with adrenal crisis, severe headaches, and visual disturbances due to significant pituitary enlargement and optic chiasm compression [20]. We treat acute, symptomatic hypophysitis according to the NCCN Guidelines with 1–2 mg/kg/d prednisolone until symptoms resolve and usually reduce the glucocorticoid stepwise to prednisolone 5 mg/d afterwards [10].

Hypophysitis often results in secondary hormonal impairment [corticotroph (80%), thyrotroph (84%), gonadotroph (76%)] [22]. The secondary hypothyroidism might resolve, but rates vary from 6 to 64%. The gonadotroph axis resolves by 12–57% [23]. Adrenal insufficiency is known to be irreversible [20]. Secondary hormonal impairment seems to improve with hypophysitis steroid therapy and often needs no replacement-therapy [20]. In our cohort, two of the four patients presenting with secondary hypothyroidism required treatment, but none had a gonadal axis insufficiency.

### Thyroid toxicity

Thyroid toxicity is a common side effect of ICI therapy. The overall incidence of hypothyroidism is about 6.6% and 2.9% for hyperthyroidism, respectively [6]. With 11% for hypothyroidism and 9% for hyperthyroidism, our study supports this data with a slightly higher incidence. According to the literature, 75% present with hyperthyroidism and 80% of them develop a secondary hypothyroidism [6]. The pathomechanism and exact etiology of thyroid dysfunction can be challenging in clinical practice, especially in combination therapies with VEGF inhibitors or in cases of pre-existing thyroid dysfunction that deteriorates during therapy [11]. In the literature, it is described that thyroid dysfunction occurs more frequently with PD-1 inhibition or combination therapy compared to anti-PD-L-1 therapy [8]. We observed a confirmative trend in our cohort. In addition to the laboratory values, a systematic symptom questionnaire is highly recommendable to reveal hypothyroidism (fatigue, increased sensitivity to cold, constipation) or hyperthyroidism (nervousness and irritability, tachycardia palpitations, tremor). If the patient is asymptomatic and TSH is not greater than 10 mIU/L, regular monitoring for hypothyroidism is sufficient. In cases of symptoms or decreased free thyroxine (fT4), substitution therapy with thyroid hormone supplementation should be initiated. For symptomatic hyperthyroidism, a therapy with β-blockers (a.e. propranolol 10–20 mg every 4–6 h) should be started. Glucocorticoids are seldom required. Thiamazole (which decreases thyroid hormone production) should only be considered in the rare case of Graves’ hyperthyroidism [10, 20].

### Primary adrenal insufficiency and insulin-depended diabetes mellitus type 1

The occurrence of adrenalitis is rare (0.7%) [10] and most likely associated with a CTLA-4 based immunotherapy [16]. The only case of adrenalitis in our cohort was seen with PD-1/CTLA-4 combination therapy. The diagnostic obstacle is to distinguish between primary adrenalitis (due to immunotherapy, bilateral adrenal metastases, or bilateral adrenal hemorrhage) and secondary adrenalitis (ICI-related hypophysitis or pituitary metastasis) [16]. Elevated ACTH and low-normal morning cortisol levels are consistent with primary adrenal insufficiency. An SST test can be helpful in the diagnostic workup. Plasma aldosterone (low) and renin levels (high) can be helpful in determining mineralocorticoid deficiency [10, 20].

For treatment, it is important to first start the replacement of corticosteroids to avoid adrenal crisis (i.e., hydrocortisone 20–0–10 mg/day and fludrocortisone 0.1 mg every other day). Patients with severe symptoms require additional fluids [10].

The incidence of a new insulin-dependent DM-type I is reported in the literature to be about 0.2% [6]. Therefore, it is no surprise that no case was seen in this study population.

It seems like immune-related diabetes is associated with PD-1 based therapy [20]. Even though, prevalence is quite rare, it’s a life-threatening complication. Patients present with polyuria, polydipsia, and weight loss due to hyperglycemia or diabetic ketoacidosis with nausea, vomiting, abdominal pain, hyperventilation, lethargy, or coma. If suspected, the workflow should include glucose level testing and evaluation for ketoacidosis using serum or urine ketones and venous blood gas evaluation. For further investigations, C-peptide and anti-islet-cell-antibodies can be determined [10, 20]. Management is based on appropriate insulin treatment for glucose control together with supportive measures [10, 20].

The study's primary strength is its cohort of patients with metastatic urothelial or renal cell carcinoma from a large urological cancer center, which provides valuable real-world data on clinical practice. However, it is important to note that the reliability of its results may be limited by the small subgroups of specific irAEs with low statistical power. For instance, the study only had one case of AI and no cases of type 1 diabetes. Despite these limitations, the research still holds clinical significance.

## Conclusion

Endocrinopathies during immunotherapy are well-manageable side effects if diagnosed correctly and treated early. However, they require close monitoring as well as good interdisciplinary cooperation with specialists in endocrinology. In this study, we were able to show that 20% of the patients treated with ICI will experience endocrinopathies, with a higher incidence and grade in the CTLA-4-based regimen. Patients on CTLA-4-based therapy have a specific risk of developing hypophysitis and should be monitored with routine fasting serum cortisone levels and standardized symptom questionnaires for irAE screening.

The total number of patients with irAEs will continue to increase over the next few years due to the broad implementation of ICI-based therapy in the treatment cascade of genitourinary cancer. Future studies should focus on the pathophysiology of irAEs and thereby identify risk factors as well as optimize strategies for monitoring and prevention to improve patients outcome.

## Data Availability

The data used to support the findings of this study are included within this article.
